# Genetic Insights from Consanguineous Cardiomyopathy Families

**DOI:** 10.3390/genes14010182

**Published:** 2023-01-10

**Authors:** Constance Maurer, Olga Boleti, Paria Najarzadeh Torbati, Farzaneh Norouzi, Anna Nicole Rebekah Fowler, Shima Minaee, Khalid Hama Salih, Mehdi Taherpour, Hassan Birjandi, Behzad Alizadeh, Aso Faeq Salih, Moniba Bijari, Henry Houlden, Alan Michael Pittman, Reza Maroofian, Yahya H. Almashham, Ehsan Ghayoor Karimiani, Juan Pablo Kaski, Eissa Ali Faqeih, Farveh Vakilian, Yalda Jamshidi

**Affiliations:** 1 Genetics Research Centre, Molecular and Clinical Sciences Institute, St George’s, University of London, Cranmer Terrace, London SW17 0RE, UK; 2 Centre for Paediatric Inherited and Rare Cardiovascular Disease, University College London and Great Ormond Street Hospital, London WC1N 1DZ, UK; 3 Department of Medical Genetics, Next Generation Genetic Polyclinic, Mashhad 009851, Iran; 4 Department of Cardiology, Faculty of Medicine, Mashhad University of Medical Sciences, Mashhad 9177948564, Iran; 5 Department of Cardiovascular Diseases, Razavi Hospital, Mashhad 9177948954, Iran; 6 Department of Pediatrics, College of Medicine, Sulaimani University, Sulaymaniyah 46001, Iraq; 7 Division of Congenital and Pediatric Cardiology, Department of Pediatrics, Faculty of Medicine, Mashhad University of Medical Sciences, Mashhad 9177948564, Iran; 8 Faculty of Medicine, Mashhad University of Medical Sciences, Mashhad 9177948564, Iran; 9 Department of Neuromuscular Diseases, UCL Queen Square Institute of Neurology, University College London, London WC1N 3BG, UK; 10 Pediatric Cardiology, King Salman Heart Center, King Fahad Medical City, Riyadh 12231, Saudi Arabia; 11 Section of Medical Genetics, Children’s Specialist Hospital, King Fahad Medical City, Riyadh 12231, Saudi Arabia

**Keywords:** cardiomyopathy, hypertrophic cardiomyopathy (HCM), dilated cardiomyopathy (DCM), genetic mutations, pathogenic variants, whole exome sequencing, consanguinity

## Abstract

Inherited cardiomyopathies are a prevalent cause of heart failure and sudden cardiac death. Both hypertrophic (HCM) and dilated cardiomyopathy (DCM) are genetically heterogeneous and typically present with an autosomal dominant mode of transmission. Whole exome sequencing and autozygosity mapping was carried out in eight un-related probands from consanguineous Middle Eastern families presenting with HCM/DCM followed by bioinformatic and co-segregation analysis to predict the potential pathogenicity of candidate variants. We identified homozygous missense variants in *TNNI3K*, *DSP*, and *RBCK1* linked with a dilated phenotype, in *NRAP* linked with a mixed phenotype of dilated/hypertrophic, and in *KLHL24* linked with a mixed phenotype of dilated/hypertrophic and non-compaction features. Co-segregation analysis in family members confirmed autosomal recessive inheritance presenting in early childhood/early adulthood. Our findings add to the mutational spectrum of recessive cardiomyopathies, supporting inclusion of *KLHL24*, *NRAP* and *RBCK1* as disease-causing genes. We also provide evidence for novel (recessive) modes of inheritance of a well-established gene *TNNI3K* and expand our knowledge of the clinical heterogeneity of cardiomyopathies. A greater understanding of the genetic causes of recessive cardiomyopathies has major implications for diagnosis and screening, particularly in underrepresented populations, such as those of the Middle East.

## 1. Introduction

Inherited cardiomyopathies, diseases of the myocardium, are a prevalent cause of heart failure and sudden cardiac death [1]. Inheritance is often complex with incomplete penetrance, high variability in disease onset and progression, and genetic heterogeneity. Providing a molecular diagnosis in patients with inherited cardiomyopathies is important for determining prognosis and management strategy as well as allowing family screening and predictions of the risk of recurrence amongst the next generation.

Hypertrophic cardiomyopathy (HCM), one of the most common inherited cardiac diseases, is defined as left ventricular hypertrophy in the absence of other causes of abnormal ventricular loading [2]. For most patients in whom a genetic cause can be identified it is associated with variants in sarcomeric genes [3]. Dilated cardiomyopathy (DCM) is characterized by left cardiac enlargement owing to left ventricular dilation as well as reduced systolic function, which is not secondary to ischemia, valvular disease, and hypertension [4,5]. In contrast to HCM, DCM has been associated with >50 different genes, including those encoding sarcomeric proteins, but also genes encoding transcription factors, the nuclear envelope, calcium handling proteins and mitochondrial proteins [6].

Most cases of HCM and DCM are inherited in an autosomal dominant manner, however recessive inheritance patterns have been reported, and often occur within populations with high consanguinity levels where there is an increased prevalence of autosomal recessive disorders [7]. In these populations, the prevalence of variants in less common (‘minor’) cardiomyopathy-associated genes is increased due to the recessive inheritance patterns but also by the presence of founder variants. Indeed, bi-allelic disease-causing variants associated with cardiomyopathies have largely been identified in individuals and families of non-European ancestry and have improved our understanding of the genetics and pathophysiology of the disease [8,9,10,11]. Importantly, recessive forms are often associated with an earlier clinical presentation (from childhood to early adulthood) and an increased risk of sudden cardiac death when compared with more common autosomal dominant forms [12,13,14]. Effective use of genetic testing in populations, such as those of the Middle East, will therefore benefit from gene panels that include genes robustly associated with cardiomyopathies in addition to ‘minor’ disease-associated genes, or alternatively whole exome/genome sequencing, which also provide an opportunity to identify novel disease associated genes. Further research in these families will improve interpretation of variants and increase our understanding of the genetic and phenotypic features of recessive forms of the disease.

Here, we describe the clinical and molecular profiles of eight consanguineous families presenting with either HCM or DCM who underwent whole-exome sequencing (WES) and in whom an autosomal recessive candidate causal variant was identified.

## 2. Materials and Methods

Detailed methodology for exome sequencing, analysis and criteria for clinical interpretation has previously been described [10]. Eight unrelated probands with HCM/DCM from consanguineous families of Middle Eastern origin were included in this study. All patients gave written informed consent, and the study was reviewed and approved by the institutional research ethics committees. Phenotypic assessment and diagnoses were based on cardiological investigations and clinical data, including echocardiography, resting and ambulatory 12-lead electrocardiogram, cardiac catheterization and cardiac MRI where available.

Exome sequencing and autozygosity mapping was performed on all probands. In brief, analysis and prioritization of discovered variants considered: (1) homozygous variants with a minor allele frequency (MAF) determined with the Genome Aggregation Database (gnomAD, https://gnomad.broadinstitute.org/; accessed on 1 April 2022), Greater Middle East Variome (GME, http://igm.ucsd.edu/gme/; accessed on 1 April 2022) and an in-house database comprising >2500 ethnically-matched individuals (SGUL MEGP) of <0.001; (2) those predicted as pathogenic by at least three of the following in silico prediction algorithms: CADD, SIFT, MutationTaster, PolyPhen2_HDIV, MutationAssessor, GERP and FATHMM; and (3) those in genes previously associated with cardiomyopathies/myopathies based on phenotypic descriptions in Online Mendelian Inheritance in Man (OMIM, https://www.ncbi.nlm.nih.gov/omim; accessed on 1 April 2022), ClinVar (https://www.ncbi.nlm.nih.gov/clinvar/; accessed on 1 April 2022) and PubMed, and those falling within a region of homozygosity. All remaining variants were validated by Sanger sequencing and co-segregation was carried out in available family members.

## 3. Results

### 3.1. Clinical Characteristics of the Patients

**Figure 1:**
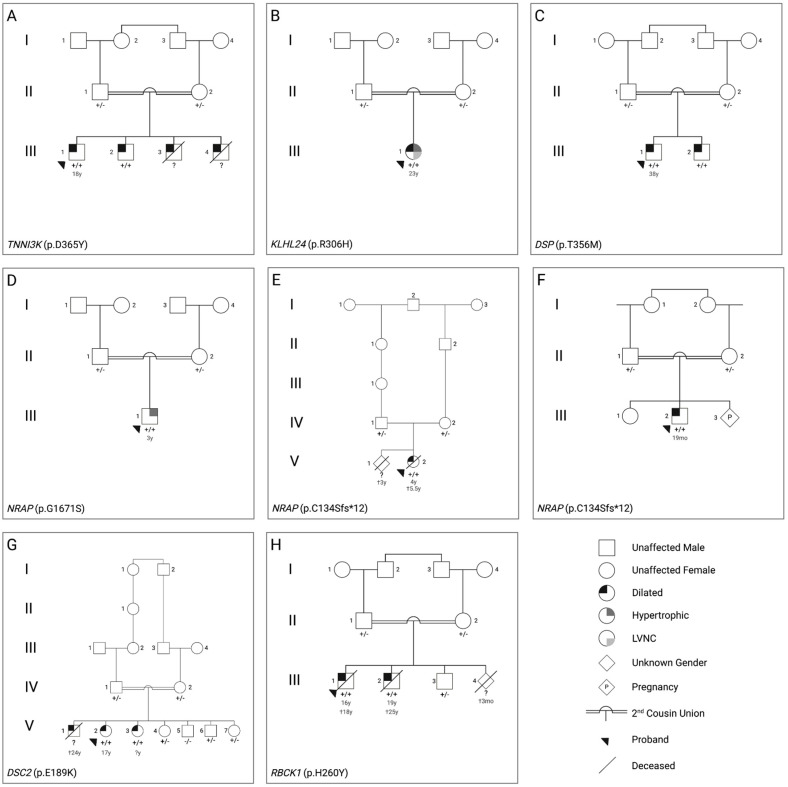
Pedigrees and candidate causal variants for the eight consanguineous cardiomyopathy families ((**A**–**H**)—see clinical characteristics/genetic findings) investigated by whole exome sequencing. Candidate gene and variant are shown for each family in the bottom left corner of each pedigree and +/− denotes genotype results from Sanger Sequencing. LVNC = Left ventricular non compaction, Dilated = Dilated cardiomyopathy, Hypertrophic = Hypertrophic cardiomyopathy. Age at presentation of symptoms and age at death (†), where available, is shown below affected individuals.

All patients were born to consanguineous parents, either first or second cousins (Figure 1) presenting with either HCM or DCM (Table 1).

In Family A, the proband is male, presenting at 18 years of age with an affected brother. On initial presentation, he had a dilated phenotype with mild LV enlargement (Left Ventricular End Diastolic Diameter (LVEDD) 55 mm), mild diastolic dysfunction but preserved systolic function (Ejection Fraction (EF) 54%) and mild mitral, tricuspid and pulmonary valve regurgitation. Over 2 years of follow up, his phenotype progressed to a mild biventricular enlargement with moderate to severe systolic (EF 35%) and diastolic dysfunction, global hypokinesia and mild left atrial enlargement. On an electrocardiogram (ECG), he had left axis deviation, intraventricular conduction delay and ST segment elevation in the anterior leads. He also had a Holter, which demonstrated ventricular ectopy (VE) (569 beats in total) with one bigeminy, one trigeminy and a non-sustained ventricular tachycardia (NSVT) of 10 consecutive beats. Both parents, who were carriers, were asymptomatic, showing no subclinical evidence of cardiac conduction system dysfunction.

The proband from Family B is female, presenting at 23 years of age with a past medical history of hyperthyroidism. On baseline echocardiogram she had severe LV dilatation (LVEDD 63 mm) with a very thick layer of non-compacted myocardium with trabeculations and recesses, overlined thin myocardial wall of apical segments and most of mid LV segments. The basal and mid inferoseptum/anteroseptum was severely hypertrophied with a maximal LV wall thickness (MLVWT) in the mid anteroseptum of 28 mm. There was concomitant mild right ventricular hypertrophy (RVH) with no outflow tract obstruction. There was severe biventricular systolic (EF 20–25%, TAPSE 16mm) and moderate left diastolic dysfunction (E/A 1.16, lateral E/E’ 13.1, septal E/E’ 19.8). There was mild LA enlargement (LA diameter 42mm), moderate mitral valve regurgitation and mild tricuspid valve regurgitation with evidence of at least moderate pulmonary hypertension (PH) (PAP 45–50 mmHg). On ECG there was right axis deviation and complete right bundle branch block (RBBB).

In Family C, the proband presented at the age of 38 years with symptoms of heart failure. On echocardiogram, there was mild RV enlargement and systolic dysfunction (TAPSE 19 mm) and normal LV size (LVEDD 35 mm) with normal systolic (EF 55–60%) and mild diastolic dysfunction (E/Ea 8/6). There was evidence of mild mitral and tricuspid valve regurgitation, with normal estimated PA pressure (30 mmHg). The ECG showed intraventricular conduction delay with repolarization abnormalities in the form of inverted T waves in all precordial leads and aVL. The proband has one affected brother.

In Family D, the proband is male, presenting at 3 years of age, due to shortness of breath and irritability. There were no affected family members. On baseline echocardiogram he was found to have hypertrophic cardiomyopathy with an MLVWT of 13 mm at the mid-septum. The LV was mildly dilated, with normal systolic and diastolic function. There was LV outflow tract obstruction secondary to systolic anterior motion (SAM) of the mitral valve, as well as mild mitral and aortic valve regurgitation. On ECG, voltage criteria were fulfilled for LVH.

Family E is from Saudi Arabia, and the proband, a young female, was symptomatic at 4 years of age with cardiac enlargement. Her early echocardiograph showed biventricular dilated cardiac enlargement, moderate-severe MR with an EF of 10% and a severely depressed LV function. Multiple cardiac failure medications were tried with minimal or absent improvement. She passed away at 5.5 years. Her parents are distant (4th or 5th) cousins with one child who passed away at 3 years of age.

Family F is also from Saudi Arabia and the proband, a young male, presented at 19 months of age with respiratory infection and cardiac failure. His echocardiography showed biventricular severe dilated CMP, severe MR with TR and decreased cardiac output. He had hypotonia with subtle facial dysmorphia. His parents are 2nd cousins with one unaffected daughter and the wife is currently pregnant with a third child.

The proband from Family G is a 26-year-old female who initially presented at 17 years of age. On baseline echocardiogram, she had a visually and mildly dilated LV (normal LVEDD 42 mm) with mild to moderate biventricular systolic dysfunction (LVEF 40–45%, RVEF 40%). On cardiac MRI her LV and RV size appeared normal, normal LV mass (69 g) with mild biventricular systolic dysfunction (LVEF 48%, RVEF 40.4%), dyskinesia in the anterior RV outflow tract free wall and the sub-tricuspid region as well as evidence of a micro-aneurysm in the mid-inferior RV wall with a transmural scar, causing late gadolinium enhancement (LGE) in the same area. Her ECG showed repolarization abnormalities in the form of inverted T waves in V2–3, flat in V4–6 and aVL. The parents were clinically unaffected and had seven offspring in total—four unaffected, one who suffered cardiac arrest at 24 years of age and a second affected daughter, two years younger than the proband.

In Family H, the proband, a young male, first presented at 16 years of age. On baseline echocardiogram there was biventricular enlargement (LVEDD 61 mm, RVEDD 24 mm) with biventricular systolic dysfunction (EF 25%, TAPSE 18 mm), mild diastolic dysfunction (E/Ea 6/7) and global hypokinesia. There was also moderate mitral and tricuspid valve regurgitation with mildly elevated PAP (35 mmHg). His ECG demonstrated intraventricular conduction delay, fulfilling voltage criteria for LV hypertrophy and repolarization abnormalities in the form of inverted T waves in V4–6 and aVL. He also underwent cardiac catheterization which showed coronary artery disease with four atherosclerotic obstructive lesions in the distal left circumflex (LCx) and right coronary artery (RCA), causing 70–90% obstruction. He was assessed for cardiac transplant and was found to be a suitable candidate, and shortly after received a transplant. There was one additional affected older brother who presented at 19 years of age who received a transplant at age 21, one unaffected brother and a sibling who passed away at 3 months of age. Despite receiving transplants both affected siblings unfortunately passed away at 25 years and 18 years of age.

### 3.2. Genetic Findings

Familial genotyping results and variant details are summarized in Figure 1 and Table 1.

In Family A, a homozygous missense variant, c.G1093T in *TNNI3K* (NM_015978), was identified. It leads to substitution of a highly conserved residue (p.[Asp365Tyr]). The variant lies in a large region of homozygosity (ROH) (30.12Mb, 97.8% homozygosity) on Chromosome 1 and is absent in the Genome Aggregation Database (gnomAD), the Greater Middle East (GME) Variome Project dataset and our in-house database (MEGP) of 2500 exomes. It is predicted pathogenic by in silico predictors including MutationTaster [disease-causing], PolyPhen-2 [damaging], and SIFT [deleterious]. An affected sibling was homozygous for the missense variant, and whilst *TNNI3K* variants are typically associated with a dominant form of cardiomyopathy, the parents were unaffected heterozygous carriers. Based on the American College of Medical Genetics and Genomics (ACMG) criteria [15], the pathogenicity of the *TNNI3K* variant was scored as a Variant of Uncertain Significance, VUS (PM2, PP1, PP3).

In Family B, a homozygous missense variant, c.G917A in *KLHL24* (NM_001349415), was identified. It leads to substitution of a highly conserved residue (p.[Arg306His]) and is located within a large ROH (23.09 Mb, 95.24% homozygosity) on Chromosome 3. The variant has an allele frequency of <0.0001 in gnomAD and GME and is found in one homozygous individual in our in-house cohort from a large multiplex cardiomyopathy family that has previously been reported [11]. Haplotype analysis of both families did not find evidence for a founder effect of this variant. Both parents are carriers of the variant. Based on ACMG criteria, the pathogenicity was scored as Likely Pathogenic (PS1, PM2, PP3, PM5).

In Family C, a homozygous missense variant, c.C1067T in *DSP* (NM_001008844), was identified. It leads to substitution of a highly conserved residue (p.[Thr356Met]) and lies in a large ROH (7.99 Mb, 96.92% homozygosity) on Chromosome 6. It is found in gnomAD at an allele frequency of <0.0001, including 1 homozygous individual, in GME at a frequency of 0.0005, and is absent in our in-house database. It is predicted pathogenic by in silico predictors including MutationTaster [disease-causing], PolyPhen-2 [probably damaging], and SIFT [deleterious]. Both parents are heterozygous carriers, and the affected sibling was homozygous for the missense variant. Based on the ACMG criteria, the *DSP* variant was scored as a VUS (PM2, PP1, PP3).

In Family D, a homozygous missense variant, c.G5011A in *NRAP* (NM_001261463.1), was identified. It leads to substitution of a highly conserved residue (p.[Gly1671Ser]). It lies in a large ROH (15.61 Mb, 97% homozygosity) on Chromosome 10. It is found in gnomAD at an allele frequency of <0.000001 and is absent in GME and our in-house database. It is predicted pathogenic by in silico predictors including MutationTaster [disease-causing], PolyPhen-2 [probably damaging], and SIFT [deleterious]. Both parents are heterozygous carriers, an unaffected sibling was homozygous for the wild-type variant, and a second unaffected sibling was a heterozygous carrier. Based on ACMG criteria, the pathogenicity score is a VUS (PM2, PP3).

In Family E, a homozygous frameshift variant, c.400_407delTGCCCAGG in *NRAP* (NM_001261463.1), was identified. The variant (p.[Cys134Serfs*12]) is absent from gnomAD, absent in GME and is also absent from an in-house Saudi matched cohort (n = 3000); however, this variant has previously been reported [16,17]. Both parents are heterozygous and unaffected. The same variant was also identified in Family F. Based on ACMG criteria, the variant is Likely Pathogenic (PS1, PM2, PP1, PP3).

In Family G, a homozygous missense variant, c.G565A, (p.[Glu189Lys]) in *DSC2* (NM_004949), was identified. It lies in a large ROH (21.67Mb, 94% homozygosity) on Chromosome 6. It is absent in gnomAD, GME and our in-house database. It is predicted pathogenic by in silico predictors including MutationTaster [disease-causing], PolyPhen-2 [probably damaging], and SIFT [deleterious]. Both parents are heterozygous carriers, as are three unaffected siblings, one unaffected sibling is homozygous for the wild-type variant, and the affected sibling was homozygous for the missense variant. Based on ACMG criteria, the pathogenicity score is VUS (PM2, PP1, PP3).

In Family H, a homozygous missense variant, c.C778T in *RBCK1* (NM_006462), was identified. It leads to a substitution of a highly conserved amino acid residue (p.[His260Tyr]) and lies in a large ROH (5.21 Mb, 97.86% homozygosity). It is absent in gnomAD, GME and our in-house database. It is predicted pathogenic by in silico predictors, including MutationTaster [disease-causing], PolyPhen-2 [probably damaging], and SIFT [deleterious]. Both parents are heterozygous carriers, as is the unaffected sibling, and the affected sibling was homozygous for the missense variant. Based on ACMG criteria, this variant is VUS (PM2, PP1, PP3).

## 4. Discussion

Inherited cardiomyopathies are genetically and clinically heterogeneous, and studies report a diagnostic yield of only 30–40% following genetic testing [18,19]. Although clinical genetic testing panels have rapidly expanded to include ‘novel’ minor genes as well as high-evidence robust genes, evidence suggests that the addition of these minor genes does not result in a demonstrable increase in clinical sensitivity and instead can result in an increased yield of variants of uncertain significance [18].

In highly consanguineous populations, such as in those of the Middle East, where there is a burden of autosomal recessive disease variants, the use of untargeted next generation sequencing approaches has successfully led to the discovery of many novel disease genes, [20,21,22,23] thereby improving our understanding of the underlying mechanisms. For many of these ‘minor’ (less common) disease genes, there is limited evidence of causality [11,14,24] and therefore a need to further validate these loci and unravel the genotype-phenotype relationships.

In this study, we investigated eight consanguineous families presenting with HCM/DCM using a whole exome sequencing approach, identifying candidate pathogenic homozygous variants in *TNNI3K, KLHL24, RBCK1, DSP*, *DSC2* and *NRAP*.

The *TNNI3K* gene encodes TNNI3-interacting kinase and is typically associated with an autosomal dominant cardiac conduction disorder with or without dilated cardiomyopathy (OMIM: 616117). In our family we identified a novel homozygous missense variant in *TNNI3K* (c.G1093T, p.[Asp365Tyr]) associated with a dilated phenotype in the affected proband, which was also present in an affected sibling. The variant lies in the Ankyrin repeat-containing domain, which is believed to play a role in protein interactions and has been demonstrated to be a protein binding target [25]. Recently a case report described a novel homozygous missense variant in *TNNI3K*, causing cardiac conduction disease in a consanguineous Pakistani family [26] and, interestingly, both heterozygous parents were unaffected as observed in the family presented here, suggesting that some *TNNI3K* variants are only disease causing when found in homozygosity. Together these results highlight an autosomal recessive form of TNNI3K-cardiomyopathy that should be considered particularly in consanguineous populations.

We recently reported homozygous missense/loss of function variants in *KLHL24*, which encodes the ubiquitin ligase substrate receptor Kelch-like protein 24, as a cause of HCM [11,16]. Gain of function variants on the other hand have been associated with skin fragility and DCM (OMIM: 617294) [16,27]. In the current study, we identify an unrelated family with the same missense variant identified in our initial report of KLHL24-HCM (c.917G>A, p.R306H), here presenting with a mixed phenotype of dilated/hypertrophic and non-compaction features. Due to the limited number of reports for bi-allelic variants in this gene, the role of missense/LOF variants is not clear. A recent study of predominantly consanguineous childhood-onset cardiomyopathy families from Saudi Arabia reported an additional case of a 14-year-old with KLHL24-HCM carrying a homozygous LOF variant (c.1161G>A, p.W387*) amongst their cohort [16]. Together, this data supports a predominantly HCM phenotype associated with bi-allelic LOF variants in *KLHL24* and supports inclusion of this gene in diagnostic panels—particularly for consanguineous populations.

Bi-allelic variants in the *RBCK1* gene, which encodes a ubiquitin ligase, have been associated with glycogen storage disease resulting in childhood or juvenile onset myopathy and a rapidly progressive dilated cardiomyopathy (OMIM: 615895) [28]. Here we identify a homozygous missense variant in *RBCK1* (c.C778T, p.H260Y) in a family with two affected siblings with DCM. In previous reports of bi-allelic RBCK1 patients experienced proximal leg muscle weakness between the age of 4–17 years of age [28]. In the family we report here, in contrast to previously reported cases, neither of the siblings presented with any signs of muscle weakness. Unfortunately, both siblings have since suffered sudden cardiac arrests (aged 18 and 25, respectively) post heart transplantation.

Bi-allelic protein-truncating variants in the nebulin-related anchoring protein gene (*NRAP*) have been identified in a few patients with severe DCM [14] but often with limited segregation data to support the findings [12,14,17,24]. In our study, we identified two unrelated Saudi families with the same homozygous frameshift *NRAP* variant (c.400_407delTGCCCAGG, p.C134Serfs*12) and a DCM phenotype. This variant has been identified in at least three additional families with DCM [16,17], which suggests it may be a founder mutation in this population. We also identified a homozygous *NRAP* missense variant in an Iranian proband in our cohort with HCM (c.G5011A, p.G1671S). Bi-allelic missense variants have not previously been reported and therefore any genotype-phenotype relationships are unclear. However, the variant is rare (gnomAD allele frequency <0.000001), lies in a large ROH (15.61 Mb, 97% homozygosity), and is predicted pathogenic by in silico predictors. Both parents are heterozygous carriers, an unaffected sibling was homozygous for the wild-type variant, and a second unaffected sibling was a heterozygous carrier. Unfortunately, there were no other affected individuals to determine further co-segregation with disease status.

Autosomal dominant and recessive variants in the desmoplakin gene (*DSP*) have been associated with arrhythmogenic right ventricular cardiomyopathy ARVC (OMIM: 607450) and biventricular DCM associated with keratoderma and woolly hair (OMIM: 605676). Here we identify a DCM family with a likely pathogenic homozygous missense variant (c.C1067T, p.T356M), which lies in the spectrin repeat domain (SRD) of desmoplakin. Only a handful of autosomal recessive *DSP* missense variants have been reported thus far, and the majority of these are associated with cardiocutaneous disorders [29,30,31]. A recent report of a Saudi DCM family with no additional skin/hair features carrying a homozygous missense variant in *DSP* (c.1459A>G, p.N487D) also in the SRD [12] further supports the pathogenicity of the T356M variant in our family without the cutaneous manifestations typically seen with recessive genotypes.

Recessive variants in *DSC2,* which encodes a desmosomal cadherin desmocollin-2, are also associated with cardiocutaneous syndromes, in contrast to heterozygous mutations, which typically cause dominant ARVC (OMIM: 610476). In our study, we identify a segregating homozygous missense variant in *DSC2* (c.565G>A, p.E189K), classified by ACMG as a VUS. Homozygous variants reported are predominantly frameshift or nonsense mutations [32,33,34,35], however a homozygous missense founder mutation (c.536A>G, p.D179G) has been described in an Italian cohort presenting with severe forms of biventricular cardiomyopathy without hair or skin abnormalities [36], which would support the pathogenicity of the E189K variant identified in our family.

Many of the variants identified in our study were classified by ACMG as a VUS, and only found in single small families, however it is likely that at least some of these are truly deleterious. Obtaining additional functional support for these variants is currently impractical and with a VUS classification they are not recommended for medical decision-making or predictive cascade testing in families. Cataloguing of variants is thus important to aid future genotype-phenotype correlations.

In summary, our findings expand the mutational spectrum of recessive cardiomyopathies and highlight the importance of recessive forms especially prevalent within populations of high consanguinity. Furthermore, we suggest the inclusion of *KLHL24*, *NRAP* and *RBCK1* as recessive disease-causing genes in diagnostic panels. Greater understanding of the genetic heterogeneity within cardiomyopathies will allow for improved interpretation of rare variants, earlier prognosis and better management of the disease, particularly in underrepresented populations, such as those of the Middle East.

## Figures and Tables

**Table 1:** Summary of clinical and genetic findings.

Family	Sex	Genotype	Phenotype	Proband Imaging	Proband ECG	Other
A	Male	*TNNI3K* (p.D365Y)	DCM	Mild diastolic dysfunction progressing to moderate/severe with global hypokinesia, LA enlargement, mild billowing of MV with trivial MR, mild TR, mild PR	Left axis deviation, criteria for LA enlargement, intraventricular conduction delay, pre-excitation	Holter: NSVT
B	Female	*KLHL24* (p.R306H)	Mixed: HCM/DCM with LVNC	Biventricular hypertrophy (LVMWT 28 mm), severe LV enlargement, severe LV systolic dysfunction, moderate diastolic dysfunction, mild LA enlargement	Right axis deviation, RBBB	
C	Male	*DSP* (p.T356M)	DCM	Mild RV enlargement, systolic dysfunction of RV, mild diastolic dysfunction, mild MR and TR	Intraventricular conduction delay, repolarization abnormalities	
D	Male	*NRAP* (p.G1671S)	HCM	Mildly dilated LV, SAM, sub-valvular AvS, mild MR, mild AR		
E	Female	*NRAP* (p.C134Sfs*12)	DCM	Biventricular dilated cardia enlargement, moderate-severe MR	EF 10%, severely depressed LV function	
F	Male	*NRAP* (p.C134Sfs*12)	DCM	Biventricular severe dilated CMP with severe MR with TR, decreased cardiac output	Not available	Hypotonia, subtle facial dysmorphia
G	Female	*DSC2* (p.E189K)	DCM	Dilated RV, mild/moderate biventricular systolic dysfunction	Repolarization abnormalities	cMRI: dyskinesia in anterior RVOT free wall and sub tricuspid region, microaneurysm in mid inferior wall, transmural scar in mid inferior of RV (LGE)
H	Male	*RBCK1* (p.H260Y)	DCM	Biventricular enlargement with biventricular systolic dysfunction, LV diastolic dysfunction, global hypokinesia, mild PAH	Intraventricular conduction delay, criteria for LV hypertrophy, repolarization abnormalities	CC: coronary artery disease (lesions in distal LCx and RCA)

LA: Left Atrium, RA: Right Atrium, LV: Left Ventricle, RV: Right Ventricle, ASD: Atrial-Septal Defect, PA: pulmonary arterial, LVEDP: Left Ventricular End Diastolic Pressure, LVNC: Left Ventricular Non Compaction, LVMWT: Left Ventricular Maximal Wall Thickness, RBBB: Right Bundle Branch Block, ccTGA: congenitally corrected Transposition of the Great Arteries, AV: atrio-ventricular, PvS: Pulmonary valve Stenosis, AvS: Aortic valve Stenosis, NAD: nothing abnormal detected, SAM: Systolic Anterior motion of the mitral valve, RVOT: Right Ventricular Outflow Tract, LGE: Late Gadolinium Enhancement, PAH: Pulmonary Arterial Hypertension, CC: Cardiac Catheter, LCx: Left Circumflex, RCA: Right Coronary Artery, MPA: Main Pulmonary Artery, PH: Pulmonary Hypertension, MR: Mitral Regurgitation, TR: Tricuspid Regurgitation, HCM: hypertrophic cardiomyopathy, DCM: dilated cardiomyopathy.

## Data Availability

Not applicable.
